# Kyphoplasty for thoracic and lumbar fractures with an intravertebral vacuum phenomenon in ankylosing spondylitis patients

**DOI:** 10.3389/fsurg.2022.962723

**Published:** 2022-07-29

**Authors:** Hao Liu, Quan Zhou, Junxin Zhang, Lei Deng, Xiayu Hu, Wei He, Tao Liu, Huilin Yang

**Affiliations:** Department of Orthopaedics, The First Affiliated Hospital of Soochow University, Suzhou, China

**Keywords:** kyphoplasty, intravertebral vacuum phenomenon, ankylosing spondylitis, vertebral fracture, PMMA

## Abstract

**Background:**

Intravertebral vacuum phenomenon (IVP) is a special sign after vertebral fractures, which is common in patients with ankylosing spondylitis (AS) and may indicate pseudarthrosis and bone nonunion that lead to spinal instability. The objective of this study is to evaluate the efficacy and safety of kyphoplasty (KP) in treating such types of vertebral fractures with AS.

**Methods:**

Sixteen patients with AS suffering from thoracic or lumbar fractures with IVP received KP from 2015 to 2020 and were monitored for more than 1 year. The visual analog scale (VAS) score was used to evaluate back pain relief. The Oswestry Disability Index (ODI) questionnaire was used to assess the improvement of the patients' living quality. The anterior and middle vertebral height restoration ratio (AVH, MVH) and the kyphotic angle (KA) were used to evaluate the radiographic results.

**Results:**

The mean follow-up period was 20.8 months (12–28 months). The VAS and ODI significantly reduced at 3 days, 3 months after surgery, and at the last follow-up compared with the preoperative outcomes (*p *< 0.05). The AVH and MVH were significantly increased compared with the preoperative outcomes (*p *< 0.05). There was a significant correction in the KA between pre- and postoperative assessments (*p *< 0.05). Asymptomatic intradiscal polymethylmethacrylate (PMMA) cement leakage was found in two patients.

**Conclusions:**

For thoracic or lumbar fractures with IVP in AS patients, KP may be safe and effective, which achieves pain relief and satisfying functional improvement, restores the anterior and middle height, and corrects the kyphotic angle of the fractured vertebra.

## Introduction

Ankylosing spondylitis (AS) is a seronegative subtype of spondyloarthropathy characterized by ossification of the facet joints, intervertebral discs, and ligaments of the axial skeleton, also known as Marie Strumpell disease or Bechterew disease (1). The prevalence of AS is reported from 0.1% to 1.4%, affecting males two to three times as often as females (2). A typical bamboo spine results from a rigid axial fused spine with progressive chronic inflammation, including sacroiliitis, spondylitis, and enthesitis (3). The axial skeleton and sacroiliac joints are primarily affected. Due to the reduced vertebral bone quality and amplified forces from the rigid spine, the risk of spinal fractures in patients with AS is higher than in the general population, especially after minor trauma (4). The data extracted from the Swedish National Hospital Discharge Registry showed that 724 patients were suffering from spinal fractures of 17,764 patients with AS during 22 years, of which 398 patients (1.4%) sustained cervical fractures (55.0%) and 302 patients sustained thoracolumbar fractures (41.7%) (5). According to statistical results from the National Inpatient Sample in 498 patients with AS, spinal fractures are present in the cervical spine (53%), thoracic spine (41.9%), lumbar spine (18.2%), and sacrum (1.5%). About 13.1% of patients suffer from multiple spinal fractures (6).

Intravertebral vacuum phenomenon (IVP), characterized by intravertebral low-density cleft, is increasingly recognized and diagnosed as a special sign after osteonecrotic vertebral fractures benefit by the advancement of radiological technology (7). It is reported that IVP is more common in AS vertebral fractures (8, 9). This can be explained by that most AS fractures are caused by hyperextension or stretch injuries, often leaving a forward opening “fish mouth” and forming a cavity in the vertebral body. Over time, the cavity can lead to the formation of pseudarthrosis and destroy spinal stability (10, 11). Treatment of this kind of fracture usually focuses on the recovery of spinal stability using the method of long-segment fixation, while the direct treatment of the early vertebral cavity is rarely reported.

With the development of minimally invasive spinal surgery all over the world, kyphoplasty (KP) has been used for the clinical treatment of osteoporotic vertebral compression fractures, spinal metastatic tumors, hemangioma, myeloma, and vertebral nonunion, which achieves pain relief, vertebral height reconstruction, and function improvement (12, 13). Although studies have reported successful treatment of spinal fracture with AS using KP, the “super-indication” usage of KP remains highly controversial (11). Moreover, KP has been applied for the treatment of osteoporotic vertebral compression fractures with IVP for several years (14, 15), but no studies have specifically used KP for the treatment of vertebral fractures with IVP in AS. In this study, we retrospectively analyzed clinical and radiological outcomes of such patient series to evaluate the feasibility, efficacy, and safety of KP when treating vertebral fractures with IVP in AS.

## Materials and methods

**Figure 1:**
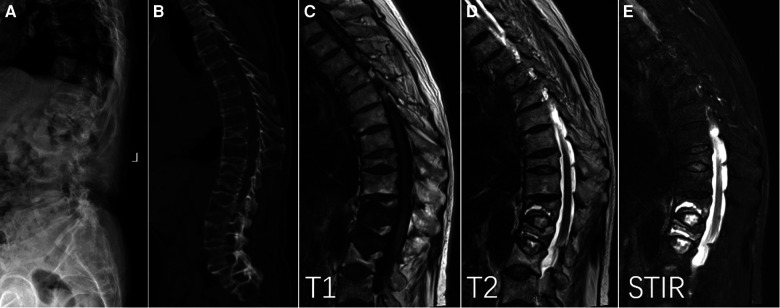
Intravertebral vacuum phenomenon (IVP) with vertebral fractures and ankylosing spondylitis. Lateral x-ray (**A**) and sagittal CT (**B**) showed low-intensity cleft throughout T12 and L1, characterized by well-defined hypointensity on T1 (**C**), hyperintensity on T2 (**D**), and hyperintensity on short time inversion recovery (**E**).

A total of 16 patients (4 females and 12 males) with AS suffering from painful thoracic or lumbar non-displaced fractures with IVP (2 levels in 2 patients, 1 level in 14 patients) received KP in our institute from May 2015 to June 2020. The mean age of the patients was 65.2 years (54–73 years). The mean duration for preoperative conservative treatment based on bed rest is 3.5 weeks (4 days to 7 months). All patients suffered from back pain or sacroiliac joint pain with morning stiffness and relieved after movement at ordinary times. The human leukocyte antigen (HLA) B27 allele was positive in all patients. Preoperative x-ray and CT scans showed a vertebral fracture, widespread vertebral osteoporosis, square vertebrae, and ossification of intervertebral discs, facet joints, and ligaments. Of the 18 fractures, 14 were Magerl A type and 4 were Magerl B type according to the AO classification of spinal fracture (16). All of the fractures were non-displaced fractures. The treated vertebral level was T11 in 2 patients, T12 in 7 patients, L1 in 6 patients, and L2 in 3 patients. The mean follow-up period was 20.8 months (12–28 months). The AS was diagnosed according to the symptoms, physical examinations, and radiologic findings such as sacroiliac joint fusion, square vertebra, and bamboo-like change. The IVP was identified according to the following diagnostic criteria (14): (1) x-ray and CT scans showed a low-intensity cleft or cavity in the vertebral body. (2) MRI showed a well-defined cavity with hypointensity on T1-weighted imaging (T1WI), isointensity or hyperintensity on T2-weighted imaging (T2WI), and hyperintensity on short-time inversion recovery (STIR) (Figure 1). Patients with previous spine surgery, infection, or tumor were excluded. The mean *T* score of lumbar spine bone mineral density was −2.8 (−2.0 to −4.5) using dual-energy x-ray absorptiomery (GE Lunar Prodigy, USA) prior to the operation. This study was approved by our Institutional Review Board.

All patients signed the consent form and received the KP procedure. After the general anesthesia was completed, the operation was performed by a bilateral transpedicular approach with trocar and cannula systems under biplanar fluoroscopic guidance. The balloon (Kyphon, Sunnyvale, CA, USA) was inserted through the cannulas and placed inside the anterior 3/4 of the vertebral body from a lateral view. The inflated balloon created a cavity in the vertebral body for the injected polymethylmethacrylate (PMMA) cement. To decrease the risk of cement leakage, the graded infusion technique (17, 18) and the incremental temperature cement delivery system (ITCDS) were used for PMMA cement infusion during the KP procedure. Under monitored continuously by fluoroscopic control in the lateral plane, the injection process was stopped if high resistance was encountered or if cement neared the posterior wall of the vertebral body. For Magerl A type fractures, patients were encouraged to walk within 3 days after the operation and received the external fixation with the lumbar or thoracolumbar bracing instrument for at least 6 weeks. For the four patients with Magerl B type fractures, bed rest for at least 2 weeks and external fixation for at least 2 months after the operation was required. Postoperative medicine therapy based on calcium and vitamin D was used for the treatment of osteoporosis. In addition, lumbar muscle exercise in the later stages of rehabilitation was suggested to strengthen the stability of the spine in every patient.

The visual analog scale (VAS) system was used to evaluate back pain relief. The Oswestry Disability Index (ODI) questionnaire was used to assess the improvement of the patients' daily life. Clinical examinations were performed before the operation, at 3 days and 3 months postoperatively, and at the last follow-up.

In the standing lateral radiograph, the mean of the measurements from the closest normal vertebral body cephalad and caudad to the treated level was used to estimate the normal height of the fractured vertebrae. The height of the fractured vertebra presented with the vertebral body height ratio calculated as follows: height ratio (%) = (compromised vertebral height/mean adjacent control vertebral height)×100. Cobb's angle was used to evaluate the correction of the kyphotic angle (KA) for the fractured vertebra.

The SPSS software system (Version 19.0; SPSS, Inc., Chicago, IL) was performed for the statistical analysis, and the results were presented as mean ± standard deviations (SD). The level of statistical significance was set at a *P* value of less than 0.05. Paired *t*-tests were used to compare within-group clinical and radiographic parameters of patients preoperatively, 3 days and 3 months postoperatively, and at the last follow-up.

## Results

The mean preoperative VAS score was 7.5 ± 1.0. After the surgery of KP, every patient achieved satisfactory pain relief. The mean VAS score significantly reduced to 2.3 ± 0.6 at 3 days after the procedure, 2.2 ± 0.7 at 3 months after the procedure, and 2.1 ± 0.7 at the last follow-up. The mean VAS score changed slightly at postoperative follow-up, which indicated the efficacy of pain control was persistent (Table 1). Similarly, the ODI scores decreased from 67.1 ± 4.4 before surgery to 27.6 ± 4.7 at 3 days after the procedure, 25.8 ± 4.9 at 3 months after the procedure, and 27.7 ± 6.5 at the last follow-up (Table 1). There was a statistically significant difference between the preoperative data and each postoperative follow-up assessment (*p *< 0.05). No neurological complications were found in all patients.

**Table 1:** Mean improvement in VAS and ODI.

	Pre-KP	3 days after KP	3 months after KP	At the last follow-up
VAS	7.5 ± 1.0	2.1 ± 0.6^a^	2.2 ± 0.7^a^	2.3 ± 0.7^a^
ODI (%)	67.1 ± 4.4	27.6 ± 4.7^a^	25.8 ± 4.9^a^	27.7 ± 6.5^a^

VAS, visual analog scale; ODI, Oswestry Disability Index; KP, kyphoplasty.

^a^ Significant difference at P < 0.05 compared with preoperation.

**Figure 2:**
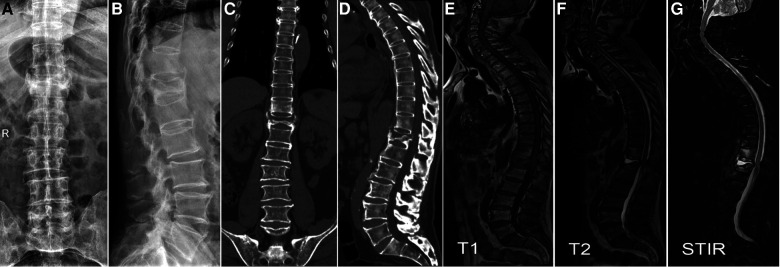
A 66-year-old male patient with T11 and T12 fractures (**A,B**). Coronal (**C**) and sagittal (**D**) CT showed intravertebral vacuum phenomenon (IVP). MRI showed well-defined hypointensity on T1 (**E**), hyperintensity on T2 (**F**), and hyperintensity on short time inversion recovery (**G**), verifying the diagnosis of IVP.

**Figure 3:**
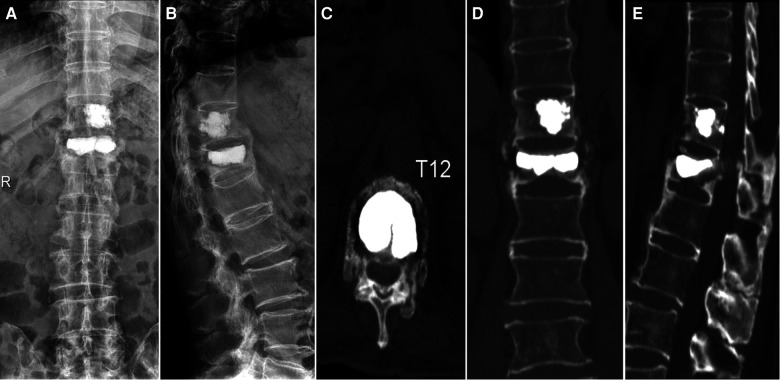
This patient received kyphoplasty. Three days later, anteroposterior (**A**) and lateral (**B**) x-ray showed the vertebral height and the Cobb's angle was restored. Traverse (**C**), coronal (**D**), and sagittal (**E**) CT showed intravertebral filling of the cavity without cement leakage.

**Figure 4:**
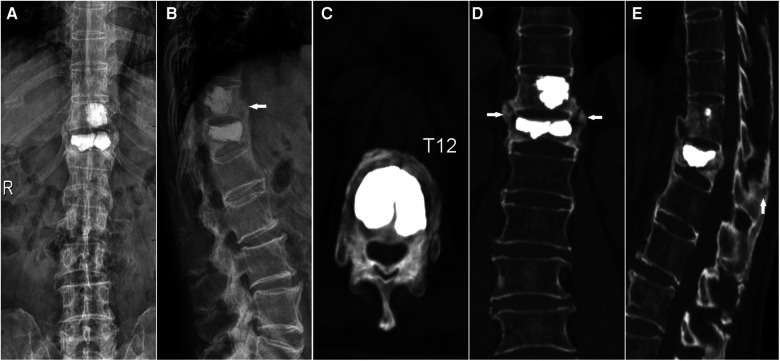
Twelve months later, bone union was observed (**A,B**). Traverse CT (**C**) showed union within the vertebra. Coronal CT showed the formation of a bone bridge (**D**). Sagittal CT showed intraspinous fusion (**E**). These ossification phenomena suggested that KP contributed to spinal stability.

**Figure 5:**
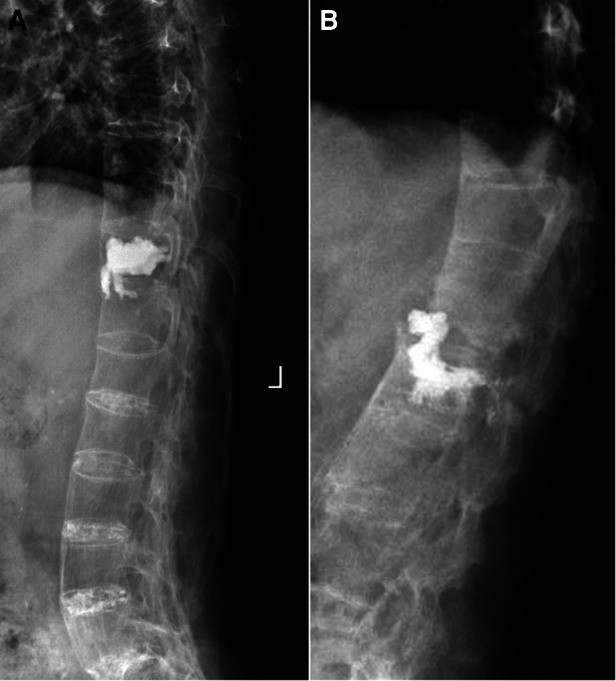
Asymptomatic intradiscal polymethylmethacrylate cement leakage was found in two patients by postoperative x-ray. The cement respectively leaked into the lower (**A**) and upper (**B**) discs of the fractured vertebrae.

The mean postoperative anterior vertebral body height (AVH) and middle vertebral body height (MVH) was significantly increased compared with the preoperative heights (*p *< 0.05) (Table 2). There was a statistically significant correction in the KA between preoperative and postoperative assessments (*p *< 0.05) (Table 2). There was no significant difference in radiographic data between the immediate postoperative and follow-up assessments (*p *> 0.05). The case of a 66-year-old male patient demonstrated typical radiographic changes after KP during a year follow-up (Figures 2–4). Asymptomatic intradiscal PMMA cement leakage was found in two patients by postoperative x-ray (Figure 5). Neither cement leakage into the spinal canal nor further dislodging of the posterior vertebral body fragments occurred.

**Table 2:** Mean restoration in the vertebral height and kyphotic angle.

	Pre-KP	3 days after KP	3 months after KP	At the last follow-up
AVH (%)	69.6 ± 11.7	82.6 ± 6.0^a^	82.8 ± 5.5^a^	83.1 ± 5.3^a^
MVH (%)	68.6 ± 11.2	79.1 ± 6.9^a^	78.9 ± 6.3^a^	79.1 ± 5.8^a^
KA (°)	20.1 ± 5.7	13.1 ± 3.6^a^	13.7 ± 3.5^a^	13.9 ± 3.3^a^

KP, kyphoplasty; AVH, anterior vertebral height ratio; MVH, middle vertebral height ratio; KA, kyphotic angle.

^a^ Significant difference at p < 0.05 compared with preoperation.

## Discussion

Gakibert and Deramond first used vertebroplasty (VP) to treat the painful vertebral hemangioma at C2 in the 1980s (19). KP that creates a cavity in the vertebral body with a balloon (inflatable bone tamp) before injecting PMMA bone cement was first designed by Wong and Reiley and got approved by the FDA for clinical use in1998. Liberman first reported the use of KP in the clinical settings in 2001 (20). With the progress of this minimally invasive surgical technique, the indications of KP are extended for many spinal diseases. A previous study in our hospital used KP to treat osteoporotic vertebral compression fractures with IVP and achieved good results (14). In this study, we focused on the application of KP in the treatment of vertebral fractures with IVP in AS.

Abnormity of osteoblastic and osteolytic remodeling along with ectopic ossification of paraspinal soft-tissue structures in AS alters the biomechanics of the spine and leads to an increased susceptibility to vertebral column fractures (1). MacMillan (21) found that traumatic fractures in patients with a partially fused spine tend to occur adjacent to the fused segments rather than through the fused region itself. The fractures in patients with AS are usually unstable three-column injuries. The risk of spinal cord injury (SCI) increases from 33% to 67% once patients with AS suffer from vertebral fractures (22, 23). Two-thirds of SCI cases are due to cervical spine fractures, and one-third are due to thoracic spine fractures (6). What is more, the mortality rate may reach 15%–30% (23). The conservative treatment based on bed rest for non-SCI cases not only makes osteoporosis worse and increases the incidence of bedsores, pneumonia, and infection but also contributes to the occurrence of vertebral nonunion (23). Open surgeries are often performed using long-segment posterior internal fixation or anterior internal fixation (24). Nevertheless, open surgeries with large trauma increase the risk of autoimmune process fatty degeneration of the paraspinal muscles and infection postoperatively. Only if SCI happens or the spinal cord is oppressed by fractural vertebral fragments, open internal fixation with decompression is suggested as the prime choice. Though percutaneous instrumentation internal fixation limits the risk of bleeding and infection due to the less muscle dissection, a high rate (10%–15%) of implant loosening occurs because of bone density within the vertebral body typically reduced in AS (22, 25).

Studies have shown that vertebral fractures with IVP had a worse prognosis than those without IVP. The formation of IVP predicts a high prevalence rate of complications such as pseudarthrosis and bone nonunion that lead to spinal instability. The use of the filling technique as VP or KP for IVP is critical to the ultimate efficacy (26, 27). In our study, PMMA cement was infused into the cavity of fractured vertebrae or pseudarthrosis to make both ends combined to reduce the pain and stabilize the spinal segments. Compared with preoperative outcomes, postoperative clinical results indicated that KP therapy successfully achieved pain relief and satisfied functional improvement in patients with AS. In addition, the radiographic results showed that KP restored the fractural vertebral height and corrected the kyphotic angle. PMMA cement is usually considered to stabilize anterior and middle columns of fractured vertebrae, which is rarely used to treat the three-column vertebral fracture. However, due to bone remodeling with ectopic ossification of posterior column spinal structures in AS, the lumbar bracing instrument for more than 6 weeks and the functional rehabilitation exercise for lumbar muscles postoperatively could provide the stable condition for osteoblastic restructure of fractured three-column vertebrae. In our study, bone union occurred not only in the vertebral body around the cement but also in paraspinal or interspinous areas during follow-up periods. These changes after KP indicates strengthened three-column stability of the spine.

Although there have been precedents that KP successfully treated Margerl type A fracture with AS (14), few reported its treatment of Margerl type B fracture. This is because Margerl type B fractures are generally considered to be unstable fractures, and KP treatment alone is not sufficient to provide adequate posterior column stability. However, in our study, four patients with Margerl type B fractures were treated with KP, and the results were satisfactory. It should be emphasized, however, that this does not mean that the indications for KP will be relaxed or that KP will become a routine treatment for unstable spinal fractures. Because all these four patients had been bedridden for at least 6 weeks (ranging from 6 weeks to 7 months) prior to receiving KP treatment, while back pain continued unabatedly. We believe that preoperative conservative treatment has a positive effect on the treatment of KP. The most significant effect of preoperative conservative treatment is absolute bed rest, which can reduce the mobility of the fractured vertebral body, reduce opening and closing, reduce the incidence of nonunion, reduce the degree of peripheral wall damage, and ultimately reduce the difficulty of surgery. However, we could not ignore the harm of conservative treatment. When these elderly patients are in the process of bed rest, they undoubtedly risk the occurrence of deep vein thrombosis in the lower limbs, lung infection, bedsores, and other serious complications. Therefore, in conservative treatment, these complications must be actively prevented and managed. Posterior open surgery was not considered the first choice due to poor physical condition. To relieve the pain, KP was performed partly as a symptomatic treatment. Although the cement filled the space in the vertebral body and played a role in stabilizing the anterior-middle column, the posterior column was not sufficiently stabilized. After surgery, therefore, the patient was required to stay in bed for at least 2 weeks and wear external fixation for at least 2 months. During the follow-up period, no complications such as vertebral dislocation, bone nonunion, or pseudarthrosis formation were observed. Based on these background factors and postoperative supportive treatment, KP exerted satisfactory efficacy.

Fractured vertebra in AS is usually along with peripheral vertebral wall damage, which increases the risk of cement leakage during KP. Furthermore, it has been reported that the risk of cement leakage increased in vertebral fractures with the vacuum phenomenon. The leakage rates were as high as 75% (28). We performed the graded infusion technique and the ITCDS to decrease the risk of cement leakage. For the fracture line in the anterior or lateral wall, small amounts of middle- or late-stage bone cement in the dough phase are first used to block the defect to prevent vertebral margin leakage. After the cement is solidified, late-stage bone cement in the paste phase or early-stage bone cement in the dough phase is injected to diffuse evenly. In addition, the viscosity–time profiles of PMMA show a strong dependence on the rate of viscosity rise along with temperature (29). Based on this characteristic, the bone cement first injected into the vertebral body is earlier solidified than that left *in vitro*. The temperature of the first injected cement is close to human body temperature (about 37°C), while the temperature of *in vitro* cement waiting for the next infusion is close to the operation room temperature (about 20°C). Therefore, waiting for about 1–2 min after the first injected cement becomes more viscous and even solidified to enhance the peripheral vertebral wall, the next cement could be injected safer with less leakage rate.

As a result, there was no cement leakage into veins or spinal canal in our study. However, it is worth noting that the incidence of disc leakage in our study was 12% (2/17), which was higher than the reported KP treatment of common compression fracture with an intravertebral cleft (14). This may be explained by the following reasons: ankylosing spondylitis discs are often ossified and joined to the upper and lower vertebral bodies as a whole, and this makes the fracture likely to involve the ossified disc. In this case, we believe that the fractured vertebra and disc should be treated as one vertebra in KP treatment. Even if disc leakage occurs, it can also be considered normal and beneficial, as it may be more conducive to filling and stabilizing the anterior and middle columns. This should be treated differently from common compression fractures.

As for many inflammatory rheumatic diseases, bone is a common target, especially vertebrae, due to its abundant blood supply. Bone loss and bone remodeling are usual changes resulting from inflammation effects in AS (30). However, the diagnosis of spinal osteoporosis may be difficult because pathologic new bone formation in syndesmophytes and periosteal bone formation interfere with the assessment of bone mineral density. The new bone formation causes an overestimation of the total bone mineral density, and the *T* values may be normal or high, even when osteoporosis is present or severe (11). Osteoporosis in patients with AS is often missed to diagnose and untreated systematically so that the complication of vertebral nonunion occurs frequently (23). Spinal fractures in patients with AS often along with osteoporosis. Sixteen enrolled patients received systematic medicine therapy postoperatively, and there were no vertebral nonunion and refracture of augmented vertebrae during the follow-up period.

The major limitation of this study was the relatively small sample size and relatively short duration of follow-up. Further studies with perspectiveness and randomization are needed to verify the results of our study.

## Conclusions

For thoracic or lumbar fractures with IVP in AS patients, KP may be safe and effective, which achieves pain relief and satisfying functional improvement, restores the anterior and middle height, and corrects the kyphotic angle of the fractured vertebra. Due to the limitation of the size of enrolled cases and the relatively short duration of follow-up, more credible results need to be deeply researched.

## Data Availability

The raw data supporting the conclusions of this article will be made available by the authors without undue reservation.
